# Systemic AAV9 gene therapy using the synapsin I promoter rescues a mouse model of neuronopathic Gaucher disease but with limited cross-correction potential to astrocytes

**DOI:** 10.1093/hmg/ddz317

**Published:** 2020-01-10

**Authors:** Giulia Massaro, Michael P Hughes, Sammie M Whaler, Kerri-Lee Wallom, David A Priestman, Frances M Platt, Simon N Waddington, Ahad A Rahim

**Affiliations:** 1 UCL School of Pharmacy, University College London, London, UK; 2 Department of Pharmacology, University of Oxford, Oxford, UK; 3 EGA Institute for Women’s Health, University College London, London UK; 4 Wits/SAMRC Antiviral Gene Therapy Research Unit, Faculty of Health Science, University of the Witswatersrand, Johannesburg, South Africa

## Abstract

Gaucher disease is caused by mutations in the *GBA* gene, which encodes for the lysosomal enzyme β-glucocerebrosidase (GCase), resulting in the accumulation of storage material in visceral organs and in some cases the brain of affected patients. While there is a commercially available treatment for the systemic manifestations, neuropathology still remains untreatable. We previously demonstrated that gene therapy represents a feasible therapeutic tool for the treatment of the neuronopathic forms of Gaucher disease (nGD). In order to further enhance the therapeutic affects to the central nervous system, we systemically delivered an adeno-associated virus (AAV) serotype 9 carrying the human *GBA* gene under control of a neuron-specific promoter to an nGD mouse model. Gene therapy increased the life span of treated animals, rescued the lethal neurodegeneration, normalized the locomotor behavioural defects and ameliorated the visceral pathology. Together, these results provided further indication of gene therapy as a possible effective treatment option for the neuropathic forms of Gaucher disease.

## Introduction

Gaucher disease (GD) is a metabolic condition and the most common lysosomal storage disorder (LSD) (1). It is caused by mutations in the *GBA* gene that encodes for the lysosomal enzyme β-glucocerebrosidase (GCase) (2). Dysfunctional or absent GCase leads to the accumulation of the substrate glucosylceramide (GluCer) and other sphingolipids in cells of the body, contributing to multisystemic manifestations.

GD has been historically classified into three distinct types based on the absence (type 1) or presence and severity of central nervous system impairment (type 2 and type 3). The visceral manifestations are common to all three types of GD (3) with a hallmark of significant hepatosplenomegaly (4) and consequent pancytopenia (5) and, frequently, lung (6) and bone disease (7). Histopathology of organs reveals the presence of the characteristic ‘Gaucher cells’ in the form of activated and engorged macrophages. A more contemporary and realistic clinical assessment considers that GD exhibits a continuum of phenotypes (1,8). For example, type 1 patients may occasionally present central or peripheral neurological manifestations (9–11). Type 2, or acute neuronopathic GD (nGD), is the most severe form with perinatal onset and subsequent death by 2–4 years of age (12). Type 3 is the chronic form of nGD, has a slower progression than type 2, and patients can live into adulthood (1,13). Neuronopathic forms of GD are characterized by perivascular and parenchymal accumulation of Gaucher cells, accompanied by severe neuronal loss in the layer IV and V of the somatosensory cortex, the cornu ammonis hippocampal regions, the thalamic nuclei of the midbrain, cerebellum, pons and medulla in the brain stem (14). Extensive astrogliosis and microglia activation further contribute to neuroinflammation and neuronal death (15), which are also reported in the nGD mouse model (16,17). The earliest neurological symptoms manifest in the first months of life with horizontal gaze palsy, hypertonic posturing, head retroflexion and swallowing difficulties (18). The severe neurodegeneration progresses rapidly, and early death usually occurs following extensive brain stem damage leading to laryngospasm and aspiration pneumonia (19).

Although enzyme replacement therapy is effective in reducing the systemic manifestations in the viscera, it is not efficacious in addressing the neurological symptoms of nGD since the recombinant enzyme cannot cross the blood–brain barrier. Therefore, there is currently no effective treatment for the type 2 or 3 neuropathology, and there is an overwhelming need to develop novel therapies.

Gene therapy is an attractive option for the treatment of nGD. We previously demonstrated that brain-targeted fetal and neonatal gene therapy using AAV9 vector rescued an acute mouse model of type 2 nGD (20). However, since the gene therapy was directed to the brain, the visceral organs continued to develop severe pathology. Using the ability of AAV9 to cross the blood-brain barrier (21–24), we administered gene therapy intravenously. All treated mice were rescued from premature death associated to neurodegeneration, and long-term improvements in visceral pathology were reported. While the results were highly encouraging given the severity of the nGD mouse model, the brain of treated mice was not completely free of pathology following neonatal intervention.

In the present study, we designed a novel vector which improves transgene expression in the central nervous system of a *Gba* knock-out K-14-lnl/lnl mouse model (25) following systemic administration to neonatal mice. Neuron-specific gene delivery under control of the strong human Synapsin I (*hSynI*) promoter has proven successful in an intracerebroventricular gene therapy pre-clinical study on another LSD characterized by severe neuropathology (26). Here we used a similar vector configuration and intravenously administered gene therapy to neonatal mice. We conducted an initial biodistribution study and analysed the safety of the new viral vector following intravenous administration. Finally, we assessed extension in life span, behavioural improvements and pathological markers in the brain and visceral organs of treated *Gba* knock-out animals.

## Results

### eGFP expression profile following intravenous administration of AAV9.hSYNI.eGFP to neonatal mice

**Figure 1:**
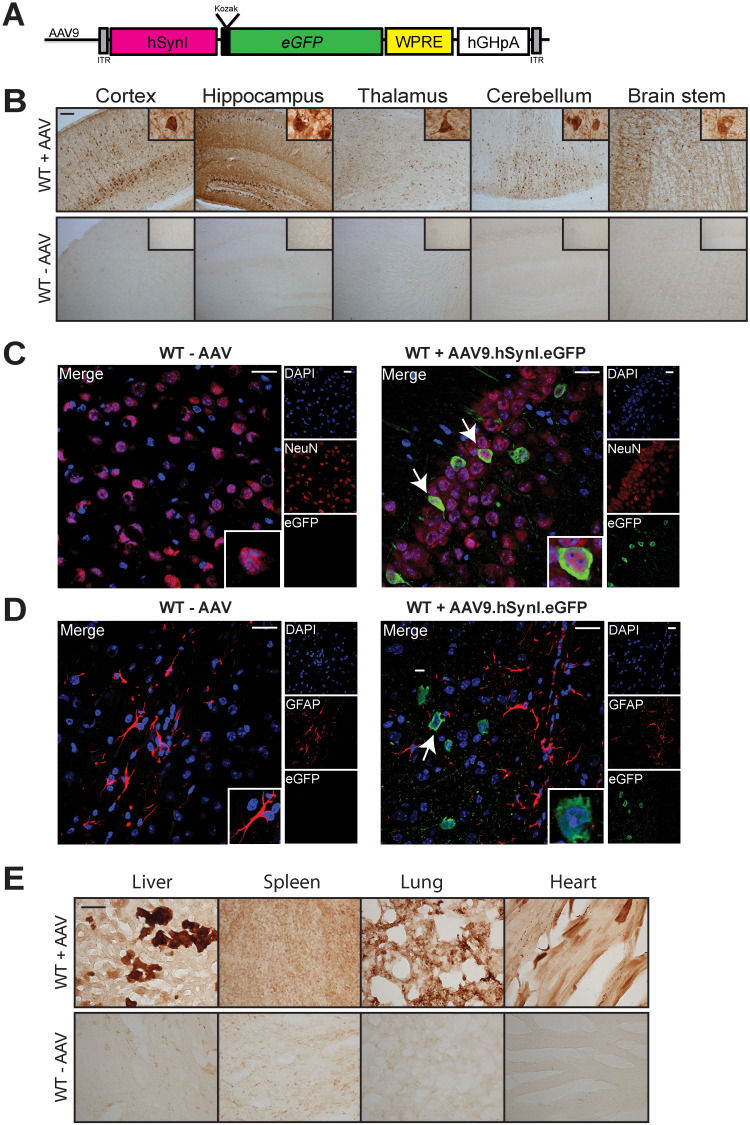
Expression profile following intravenous administration of 2 × 10^11^ vg AAV9.hSYNI.eGFP to neonatal mice. (**A**) Representation of the single-stranded AAV9 vector used in the biodistribution study. *eGFP* gene expression was driven by the human synapsin I promoter (*hSynI*). WPRE, enhancer woodchuck hepatitis virus post-transcriptional regulatory element; hGHpA, human growth hormone polyA; ITR, inverted terminal repeat. (**B**) Administration of the vector resulted in extensive rostro-caudal eGFP expression in the brain of injected mice following systemic administration (WT + AAV). Scale bar: 100 μm. Brain sections were co-stained for the GFP reporter and neuronal marker NeuN (**C**) or astrocytic marker GFAP (**D**). Confocal microscopy imaging confirmed neuronal-specific tropism of the vector in brain sections from injected mice (white arrows in WT + AAV9.hSYNI.eGFP). Scale bars: 20 μm. (**E**) Strong transgene expression was detected in the liver of injected mice (WT + AAV). Transduction of spleen, lung and heart was also observed. Scale bar: 100 μm.

We first conducted experiments to assess the expression profile of an AAV9 vector that drives the expression of the *eGFP* reporter gene under control of the neuron-specific *hSynI* promoter. This vector also included the human growth hormone polyA and woodchuck hepatitis virus post-transcriptional regulatory element (WPRE) sequences (Fig. 1A). Three wild-type mice were injected into the temporal vein at day of birth with 2 × 10^11^ viral vector genomes (vg) of AAV9.hSYNI.eGFP. Three uninjected littermates were used as negative age-matched controls. The animals were sacrificed at post-natal day 30 (P30), and eGFP expression analysis was carried out on the harvested tissues.

**Figure 2:**
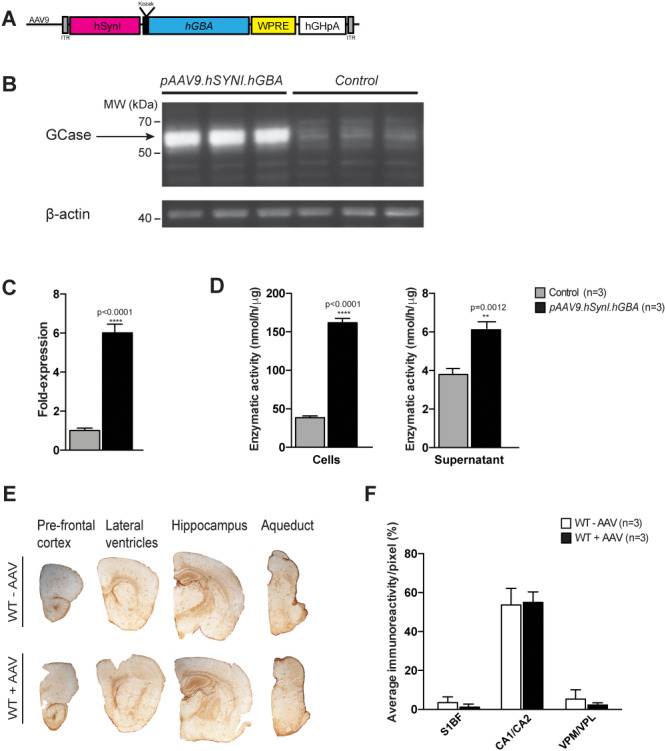
*In vitro* testing of the *pAAV9.hSynI.GBA* plasmid and assessment of neuroinflammatory response to the AAV9.hSYNI.hGBA vector in wild-type animals. (**A**) Schematic of the AAV9.hSYNI.hGBA expression cassette, where *hGBA* represents the human *GBA* gene. (**B** and **C**) Western blot analysis and protein quantification following transfection with the *pAAV9.hSynI.GBA* plasmid showed increased expression of GCase protein compared to controls. (**D**) Enzymatic assay on cell lysate and supernatant demonstrated that GCase was enzymatically functional, with increased activity in transfected samples. (**E**) Assessment of neuroinflammation following systemic administration of AAV9.hSYNI.hGBA to neonatal wild-type mice. Immunohistochemical staining for the marker GFAP did not show increase in astrogliosis in brains from injected mice (WT + AAV) in different regions compared to controls (WT—AAV). (**F**) Staining quantification confirmed normal GFAP levels in cortex (S1BF), hippocampus (CA1/CA2) and thalamus (VPM/VPL) in injected mice (WT + AAV).

Brain sections of injected and uninjected mice were stained with antibody against eGFP, and light microscopy analysis was performed. The systemic administration of the viral vector resulted in widespread transduction throughout the whole brain (Fig. 1B). Intense eGFP expression was detected in areas of particular relevance to nGD including layer V of the cortex, the dentate gyrus and CA1/CA2 region in the hippocampus, the thalamus, cerebellar lobules and in the gigantocellular nuclei region in the brain stem. Higher magnification light microscopy images in Figure 1B revealed that transduced cells appeared to have a neuronal morphology. To confirm that *eGFP* gene expression was specific to neurons and not other glial cells, brain sections were co-stained with fluorescent antibodies for eGFP and the neuronal marker NeuN and counterstained with the nucleic acid marker DAPI (Fig. 1C). In the brains of injected mice, it was possible to identify NeuN-positive cells expressing the eGFP protein (white arrows). Brain sections were also stained with antibody for the glial fibrillary acidic protein GFAP (Fig. 1D). eGFP expression (white arrow) was not present in any cells identified as GFAP-positive, confirming the neuron-specific expression profile of the AAV9.hSYNI.eGFP vector.

Interestingly, an examination of various visceral organs from administered mice revealed extensive eGFP expression compared to control unadministered mice. This was most prominent in the liver, but also visible in the spleen, lung and heart (Fig. 1E). Therefore, the *hSynI* promoter in the context of this AAV backbone mediates not only neuron-specific expression in the brain but also expression in various visceral organs, which is sustained through rapid growth from the neonatal to adult phase of murine development.

### *In vitro* functionality assessment of AAV9.hSYNI.hGBA and evaluation of neuroinflammatory response in wild-type mice

Having confirmed robust *hSynI-*mediated *eGFP* expression in neurons of the murine brain and also cells of visceral organs, we replaced the transgene with the human *GBA* cDNA sequence (*pAAV9.hSynI.GBA*) (Fig. 2A). To confirm whether correct full length GCase protein was expressed, HEK-293 T cells were transfected with the plasmid described in Figure 2A. Untransfected cells were used as a control. A western blot analysis using antibodies against GCase and β-actin as loading control was performed on cell lysates. Cells transfected with the *pAAV9.hSynI.GBA* plasmid produced supraphysiological levels of human GCase of the correct size protein (≈60 kDa) when compared to the physiological levels in untransfected cells (Fig. 2B). Subsequent quantification of the relative protein expression revealed that transfected cells expressed 6-fold higher GCase than untransfected controls (Fig. 2C). An enzymatic activity assay was then performed to confirm that the human GCase was functional. This showed that the enzymatic activity of GCase extracted from transfected cells was 5-fold higher than the endogenous levels in untransfected cells (Fig. 2D). The supernatant was also tested in order to demonstrate that functional GCase was secreted from cells. GCase enzymatic activity in the supernatant was higher in the transfected samples than in the controls.

To evaluate potential toxicity from administration of a high dose of the AAV9.hSYNI.hGBA vector, CD1 wild-type mice were intravenously injected with 2.4 × 10^12^ vg of the viral vector on the day of birth. The animals were sacrificed after 1 month and the brains were assessed for the presence of a neuroinflammatory response. Sections of brain from injected mice (WT + AAV) and uninjected animals (WT—AAV) were examined by immunohistochemistry using an antibody against the astrocytic marker GFAP (Fig. 2E). We did not observe any hypertrophy or activation of astrocytes in any region of the brains from administered mice. The level of immunoreactivity in the barrel field of the somato-sensory cortex (S1BF), cornu annuli 1 and 2 of the hippocampus (CA1/CA2) and the ventral posteromedial and posterolateral nuclei of the thalamus (VPM/VPL) was measured (Fig. 2F). Quantification of GFAP staining confirmed that the staining intensity was comparable to the uninjected age-matched littermate controls, with no statistical difference between injected and uninjected mice.

### Extension of lifespan and assessment of neurological manifestation in K-14-lnl/lnl mice treated with AAV9.hSYNI.hGBA

**Figure 3:**
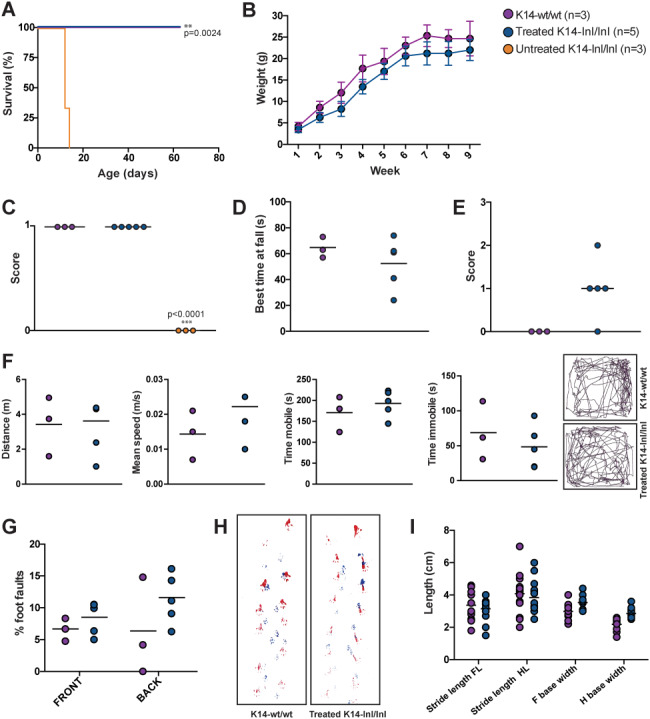
Gene therapy prolonged life span of *Gba* knock-out mice and normalized neurological manifestations. (**A**) Kapan–Meier survival curve. Untreated *Gba* knock-out animals (K14-lnl/lnl) reached their humane end-point at 2 weeks of age. Intravenous administration of AAV9.hSYNI.hGBA resulted in extended life of treated K14-lnl/lnl mice. Treated mice and wild-type controls (K14-wt/wt) were sacrificed at 9 weeks of age. (**B**) Weekly body weight of treated K14-lnl/lnl mice and age-matched K14-wt/wt controls. (**C**) Righting reflex test. P14 untreated K14-lnl/lnl mice were unable to right themselves when placed in supine position (score 0), while P14 treated mice showed fast latency to turn (score 1). (**D**) Best time at fall in rotarod test. P66-treated mice did not show loco-motor impairment when positioned on the accelerating rotarod. (**E**) Hind limb clasping test. P66-treated mice showed different clasping phenotypes, from moderate clasping (score 2) to normal behaviour (score 0). (**F**) Open field test (data analysis and traces). Mice were assessed for distance walked, average speed, time of mobility and immobility when placed into the open field box. P66-treated K14-lnl/lnl mice did not show significant differences to the age-matched controls. (**G**) Foot fault test. Paw misplacement was counted over the total number of steps, with P66-treated mice showing no difference compared to wild-types. (**H** and **I**) Traces and data analysis of footprint test. Indicative gait analysis measuring stride length and base width did not show significant differences between P66-treated K14-lnl/lnl mice and wild-type controls.

Having shown that neonatal gene delivery was effective and overexpression of GCase did not promote neurotoxicity in the brain of injected animals, 2.4 × 10^12^ vg of the AAV9.hSYNI.hGBA vector was administered intravenously to five *Gba* knock-out mice (K14-lnl/lnl) on the day of birth. Age-matched wild-type mice and untreated knock-out mice were used as controls. Consistent with previous studies, untreated knock-out mice developed progressive neurological symptoms at 10 days after birth and reached their humane end-point at 2 weeks of age, at which point they displayed advanced tetraparesis and seizures. The administration of gene therapy resulted in a significant extension of the lifespan of all treated knock-out mice (Fig. 3A). Treated knock-out and wild-type mice were sacrificed at 9 weeks of age. For the duration of the experiment, the body weight of the animals was monitored weekly (Fig. 3B). Treated K14-lnl/lnl mice gained weight throughout their lifespan, although there was a trend towards them being smaller than the age-matched controls, albeit not statistically significant.

Neurological manifestations in treated mice and wild-type controls were assessed by a series of behavioural tests. K14-lnl/lnl mice exhibited motor dysfunction and paralysis at 2 weeks of age, where they were completely unable to return to prone position when placed in supine position. At P14, untreated knock-out mice failed the self-righting reflex test (score 0), while all treated mice and wild-type controls were significantly better at being able to right themselves in less than 1 s (score 1) (Fig. 3C). At this stage, untreated animals were too severely affected and could not undergo any further neurobehavioural assessment. At 60 days of age, treated knock-out mice did not show obvious symptoms of loco-motor dysfunction when placed on the rotarod apparatus, although the average time at fall was slightly shorter than the littermate controls, but not statistically significantly so (Fig. 3D). Cerebellar ataxia was assessed by monitoring limb clasping phenotype (Fig. 3E). While K14-wt/wt controls extended all four limbs when suspended by the tail (score 0), P66-treated mice showed a diverse phenotype. One animal displayed severe clasping on both limbs (score 2), three mice showed a mild hind limb clasping behaviour and one mouse did not show any clasping phenotype comparable to wild-types. An open field analysis showed that P66-treated K14-lnl/lnl mice performed equally to wild-type littermates in terms of distance travelled, average speed, time in motion and time immobile (Fig. 3F). From the movement traces it was possible to observe that treated animals showed exploratory behaviour and did cross the centre of the chamber several times, comparable to the wild-type controls. Motor coordination was also assessed by counting the number of paw misplacements over 1 min when P66 animals were free to walk on a grid (Fig. 3G). Both treated knock-out mice and wild-type controls missed a comparable number of paw placements, showing no significant difference. Footprint analysis (Fig. 3H) was used to evaluate gait in treated and control mice at 9 weeks of age. Stride length and base width were measured (Fig. 3I), demonstrating that treated K14-lnl/lnl mice did not show ataxic movement, averagely maintaining regular-base gait and constant step length.

### Neuropathology analysis of brains from AAV9.hSYNI.hGBA-treated K14-lnl/lnl mice

**Figure 4:**
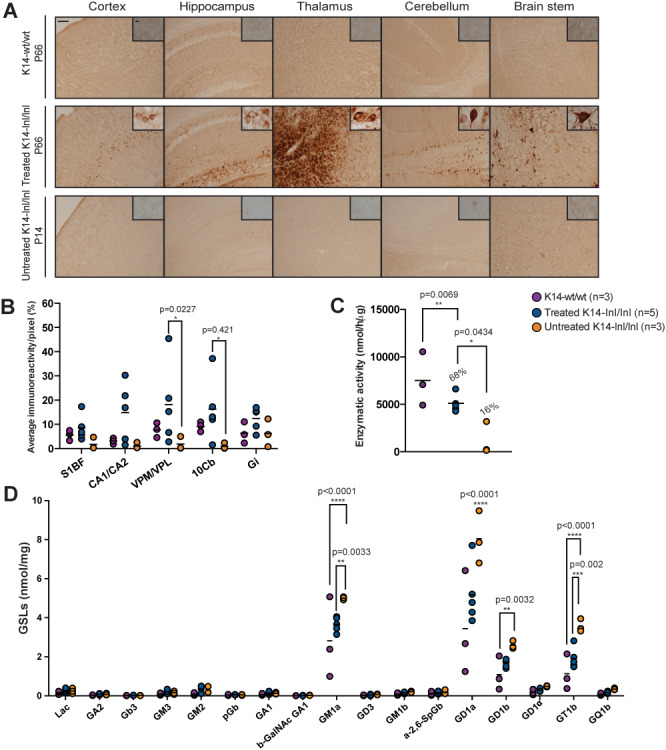
Overexpression of GCase in brain from treated K14-lnl/lnl mice. (**A**) Brain sections from wild-type, treated and untreated K14-lnl/lnl mice were stained for GCase. Scale bars: low magnification 100 μm; high magnification 60 μm. (**B**) Quantification of immunoreactivity in cortex (S1BF), hippocampus (CA1/CA2), thalamus (VPM/VPL), cerebellum (10Cb) and brain stem (Gi) confirmed widespread expression of GCase in injected K14-lnl/lnl mice. (**C**) Gene therapy resulted in increased GCase enzymatic activity in the whole homogenate brain of treated mice. (**D**) GSL analysis on brain homogenates. GM1a, GD1a and GT1b levels were elevated in P14 K14-lnl/lnl mice compared to both P66 wild-type controls and P66-treated mice.

At 66 days of age, treated K14-lnl/lnl mice and age-matched wild-type controls were sacrificed, and immunohistological analysis was performed. Brain sections of controls, treated knock-out and untreated mice were stained with an antibody against the GCase protein, and the most affected regions observed in Gaucher patients were analysed (Fig. 4A). Widespread AAV9.hSYNI.hGBA-mediated expression of GCase was detected in neurons throughout the whole brain of injected mice, particularly in the cortex, hippocampus, thalamus, cerebellum and brain stem. Quantification of immunoreactivity confirmed that systemic administration of the vector resulted in increased GCase expression compared to untreated affected mice, especially in the thalamic nuclei VPM/VPL and the cerebellar lobe 10Cb (Fig. 4B). GCase enzymatic activity was measured in frozen brain homogenates (Fig. 4C). Enzymatic activity in the brain of treated knock-out mice was on average 68% of wild-type levels, but statistically there was no significant difference between the two groups, while end-stage untreated mice retained around 10–16% of residual enzymatic activity consistent with what was previously shown in (25). Glycosphingolipid (GSL) profile analysis was also conducted on brain homogenates (Fig. 4D). Most GSL species were not elevated in young P14 knock-out mice and in older P66 treated mice. However, GM1a, GD1a and GT1b levels were significantly elevated in young untreated P14 knock-out brains compared to both older P66 wild-type and P66 treated knock-out mice. GD1b was elevated in untreated *Gba* knock-outs compared to wild-type controls, but there was no significant difference with treated K14-lnl/lnl mice.

**Figure 5:**
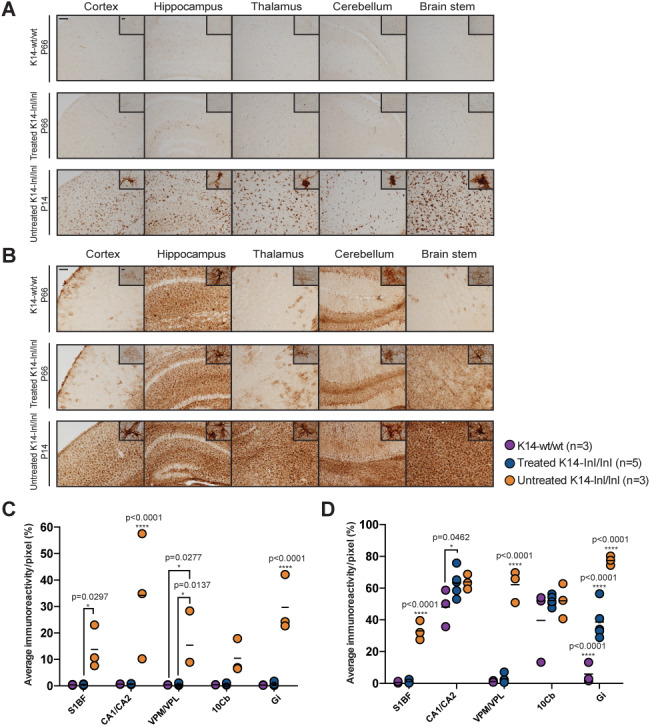
Reduction of microglia activation and astrogliosis in treated K14-lnl/lnl mice. (**A**) Brain sections stained for the microglial marker CD68 and (**C**) quantification of immunoreactivity in cortex (S1BF), hippocampus (CA1/CA2), thalamus (VPM/VPL), cerebellum (10Cb) and brain stem (Gi). Gene therapy normalized microglia activation to wild-type levels in P66-treated K14-lnl/lnl mice. Scale bars: low magnification 100 μm; high magnification 60 μm. (**B**) Immunochemical staining of brain sections for the astrocyte marker GFAP and (**D**) quantification of immune reactivity in cortex (S1BF), hippocampus (CA1/CA2), thalamus (VPM/VPL), cerebellum (10Cb) and brain stem (Gi). Gene therapy partially reduced neuroinflammation in cortex, thalamus and brain stem of K14-lnl/lnl treated brains. Scale bars: low magnification 100 μm; high magnification 60 μm.

Brain sections underwent additional immunohistochemical staining using the Cluster of Differentiation 68 (CD68) marker (Fig. 5A). Intense and diffuse staining was observed in brains of P14 untreated K14-lnl/lnl mice. Higher magnification light microscopy revealed hypertrophic activated microglia morphology. Microglia activation was normalized in brains of P66 treated mice; CD68-positive staining was similar to that in brain sections from age-matched controls. Higher magnification of microglia in brain sections from treated mice also revealed a resting morphology identical to those observed in control wild-type mice. This result was corroborated by quantification of immunoreactivity (Fig. 5C). In all analysed brain regions, CD68 staining was significantly elevated in untreated knock-out mice compared to both treated mice and wild-type controls. Quantification revealed no significant difference in CD68 staining between treated and age-matched wild-type controls, indicating sustained prevention of any microglia-mediated inflammatory response.

The GFAP marker was used to assess an astrocyte-mediated inflammatory response (Fig. 5B). Examination of brain sections from P14 untreated K14-lnl/lnl mice showed extensive and diffuse astrogliosis in all areas examined and higher magnification revealed hypertrophy of labelled cells. The sections from treated K14-lnl/lnl mice exhibited a normalization of the GFAP immunopositivity and were comparable to sections from wild-type mice, with the exception of the brain stem. In this region, there was a reduction in the inflammatory response but it did not appear to be comparable with wild-type levels. These observations were partially supported by quantification of staining showing that the administration of the AAV9.hSYNI.hGBA vector resulted in significant reduction of neuroinflammation in treated mice in the S1BF cortical region and the VPM/VPL thalamic nuclei compared to untreated knock-out animals and no significant difference from wild-type animals (Fig. 5D). However, in the CA1/CA2 regions of the hippocampus and the Gi region of the brain stem, the levels of GFAP staining were still significantly higher than those measured in control wild-type mice.

**Figure 6:**
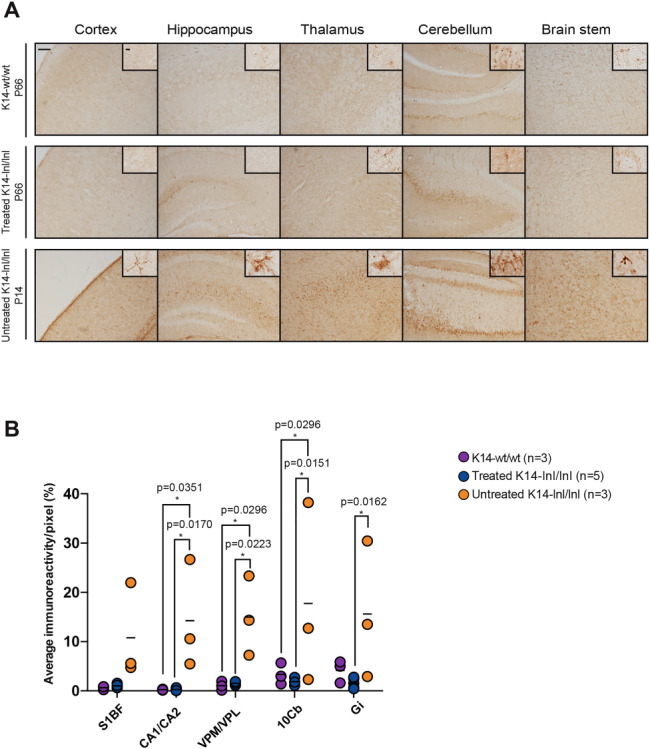
Normalization of lysosome accumulation following gene therapy administration. (**A**) Brain section images and (**B**) quantification of the staining for the lysosomal marker LAMP1. Treated brains showed significant reduction in lysosome accumulation in several brain regions. Cortex (S1BF), hippocampus (CA1/CA2), thalamus (VPM/VPL), cerebellum (10Cb) and brain stem (Gi). Scale bars: low magnification 100 μm; high magnification 60 μm.

Sections were also stained for the Lysosome-Associated Membrane Protein 1 (LAMP1) (Fig. 6A). Lysosomal accumulation is observable in the brain of K14-lnl/lnl mice in all regions examined. This was not the case in the brains from treated mice that were comparable to those from wild type controls. Quantification of the immunoreactivity (Fig. 6B) confirmed the significant increase in LAMP1 staining in all brain regions in brain sections from K14-lnl/lnl, while the lysosomal marker levels were completely normalized to wild-type level in all analysed brain regions of treated mice.

### Gene therapy prevents neuronal loss in treated K14-lnl/lnl brains

**Figure 7:**
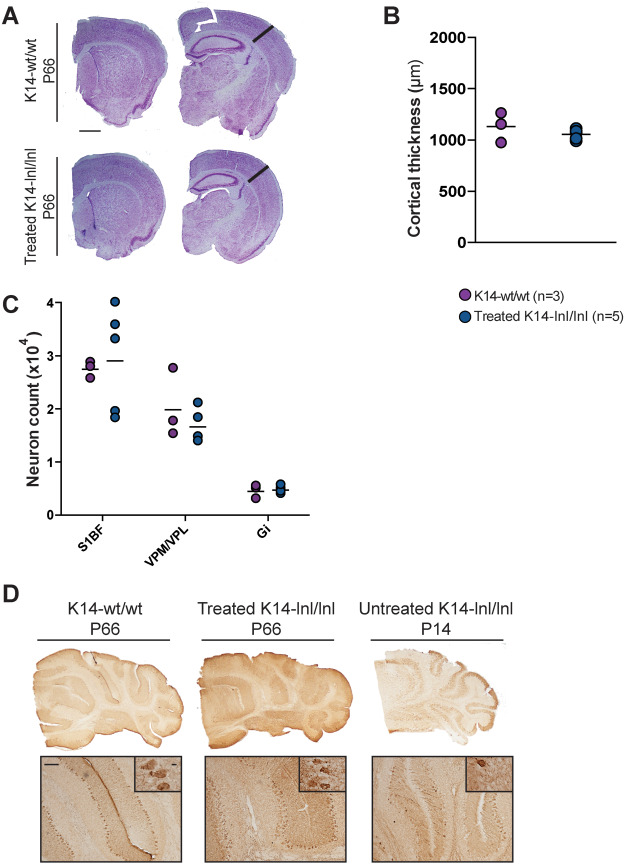
Stereology analysis on brain sections of P66-treated mice and age-matched controls. (**A**) Nissl staining of brain sections from wild-type and treated K14-lnl/lnl mice. Scale bar: 1 mm. (**B**) Thickness measurement of the S1BF cortical region showed no difference between treated K14-lnl/lnl mice and controls. (**C**) Neuron counting in cortex (S1BF), thalamus (VPM/VPL) and brain stem (Gi). Gene therapy prevented cell loss in 9-week-old treated mice. (**D**) Non-quantitative analysis of Purkinje neuron loss in the cerebellum via calbindin staining. Scale bars: low magnification 100 μm; high magnification 60 μm.

We have previously demonstrated that the P12 *Gba* knock-out mice present reduced cortical thickness and significant neuron loss in different brain regions (20). Therefore, brain sections from P66-treated mice and wild-type controls were stained with Nissl coloration and a stereological analysis was performed in order to assess brain atrophy and neuronal counts in age-matched animals. Macroscopic examination of the anatomical architecture by light microscopy did not highlight any obvious abnormalities (Fig. 7A). Measurements of cortical thickness in the S1BF region demonstrated no differences between treated K14-lnl/lnl mice and age-matched wild type controls (Fig. 7B).

In order to establish whether the treatment with AAV9.hSYNI.hGBA ameliorated the neurodegeneration in treated mice, neuronal counts were performed using the stereological technique combining optical dissector and fractionator. The number of neurons was evaluated in the S1BF cortical region, the VPM/VPL thalamic nuclei and the Gi region in the brain stem (Fig. 7C). Neuron counts in brains harvested from P66 treated knock-out mice were comparable to age-matched wild-type controls in all analysed regions, demonstrating that gene therapy prevents neuronal loss in treated animals.

A non-quantitative analysis of Purkinje neurons in the cerebellum was conducted. Cerebellar sections were stained using the Purkinje neuron-specific marker calbindin, and light microscopy images were taken (Fig. 7D). The examination of high magnification images revealed that there was no evident Purkinje cell loss in cerebella from P66 treated knock-out mice, while fewer neurons were detected in the tissue of P14 end-stage untreated K14-lnl/lnl animals.

### Effects of gene therapy on the visceral pathology

**Figure 8:**
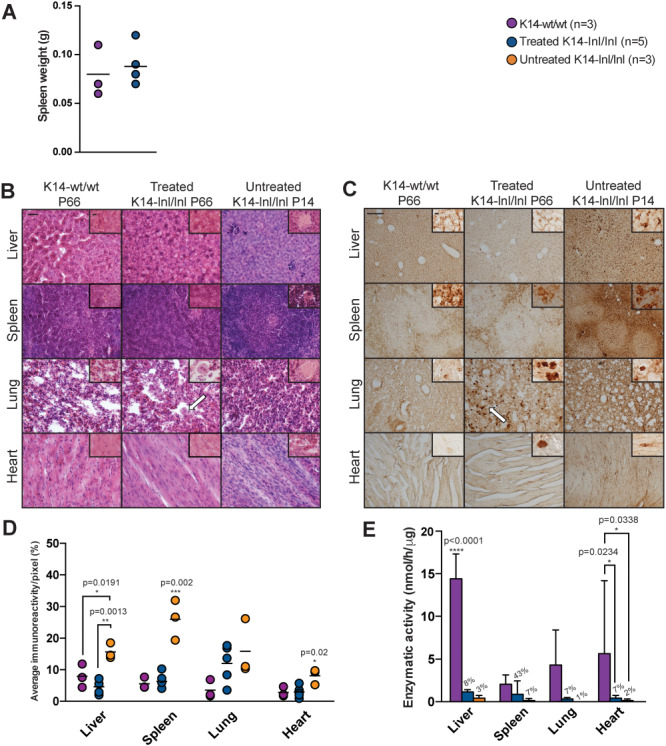
Analysis of visceral pathology following gene therapy administration. (**A**) Spleen weight of P66-treated K14-lnl/lnl mice and wild-type controls. (**B**) Haematoxylin and eosin staining of liver, spleen, lung and heart sections. White arrows indicate Gaucher cells. Scale bars: low magnification 200 μm; high magnification 100 μm. Immunohistochemical staining for the macrophagic marker CD68: (**C**) light microscopy images and (**D**) quantification. White arrows indicate Gaucher cells. Scale bars: low magnification 200 μm; high magnification 100 μm. (**E**) GCase enzymatic activity from tissue homogenates. Residual activity for treated and untreated K14-lnl/lnl animals is also indicated as percentage of wild-type levels.

We have previously shown that an intravenous administration of an AAV9 vector expressing *GBA* under the control of the ubiquitous *GUSB* promoter can ameliorate visceral pathology in the K14-lnl/lnl mouse model. Since evidence of *hSynI*-mediated gene expression within visceral organs and cells of non-neuronal lineage has been shown, amelioration of the visceral pathology following intravenous gene therapy was evaluated. Organomegaly is one of the most common systemic manifestations in patients and is recapitulated in the long-term intracerebroventricular treated mouse model (20). Gene therapy prevented enlargement of the spleen in 9-week old treated mice with no significant difference compared to measurements from wild type mice (Fig. 8A).

Sections from the liver, spleen, lung and heart were stained with haematoxylin and eosin dye (Fig. 8B) to evaluate tissue architecture and the presence of Gaucher cells. High magnification images of organs from P14 untreated knock-out mice revealed the presence of engorged macrophages. Gene therapy prevented the accumulation of macrophages in liver, spleen and heart tissue of P66 treated knock-outs, although some enlarged Gaucher cells were present in the lung sections.

Light microscopy images (Fig. 8C) showed reduction in accumulation of CD68-positive cells in the liver and spleen of P66 treated animals compared to P14 untreated knock-outs. Gene therapy did not completely ameliorate the pathology in the lungs, as areas of activated macrophages were identified in the tissue of P66-treated animals. A few scattered CD68-positive cells were identified in the heart of treated K14-lnl/lnl mice. The estimation of the immunoreactivity confirmed that gene therapy significantly reduced CD68 staining in liver, spleen and heart of treated mice compared to younger untreated controls (Fig. 8D). A decrease in macrophage infiltration was observed in lung tissue of treated knock-outs compared to untreated mice although this was not statistically significant.

GCase enzymatic activity remained significantly lower in liver, lung and heart samples from treated mice, in which GCase activity was less than 10% of wild-type levels (Fig. 8E). Spleen from treated animals had 43% of enzymatic activity compared to wild-type controls. Untreated K14-lnl/lnl mice had residual enzyme activity ranging between 1 and 7% of wild-type levels in all visceral organs. Although the enzymatic activity in tissues harvested from treated mice was not normalized to wild-type levels, GCase activity was greater in P66-treated animals than younger *Gba* knock-out untreated mice.

## Discussion

Viral gene therapy offers the unique opportunity to obtain long-lived therapeutic effects, particularly for neurological diseases where the treatment needs to reach restricted tissues such as the brain and achieve high levels of transduction (27). Because of its ability to cross the blood-brain barrier following systemic administration (21), AAV9 has been widely used for gene delivery to the central nervous system in several preclinical studies (28) as well as in recent clinical trials for other LSDs (NCT02716246 and NCT03315182) and spinal muscular atrophy (NCT03505099) (29).

We have previously demonstrated that an acute mouse model of nGD can be rescued long-term from neurodegeneration-associated death by a single intravenous administration of an AAV vector at birth (20). While encouraging, the neuropathology was not completely prevented. Here we designed a gene therapy vector with a strong neuron-specific synapsin I promoter to improve gene expression in the central nervous system following systemic administration to this mouse model of nGD.

Firstly, we conducted a systemic gene expression study using the eGFP reporter driven by the synapsin promoter. Neonatal intravenous administrations of the viral vector resulted in widespread and intense neuron-specific expression in the brain of injected mice. Surprisingly, systemic gene delivery of the neurotrophic vector resulted in eGFP expression in non-neuronal cells in visceral organs, such as the liver and spleen. Having confirmed the potential use of the vector and established that overexpression of GCase in the brain did not cause neurotoxicity, we proceed in administering gene therapy to neonatal *Gba*-defective mice. The treatment resulted in significant extension of life span, prevented weight loss and ameliorated indices of locomotor and behavioural deficits.

The K14-lnl/lnl model is one of the most aggressive mouse models of neurovisceral metabolic diseases, the treatment of which requires an early intervention to rescue it from death at 12–14 days of age. The ability of the vector to rescue the nGD mice given the short window of therapeutic intervention, even when administered at birth, is encouraging particularly if taking into consideration that the viral vector used carried a single-stranded DNA genome. The conversion into double-stranded DNA is necessary to commence gene expression and is considered to be a limiting factor that influences expression time and rate mediated by ssAAV vectors (30). In spite of the presence of neuropathology already on the day of birth (20), AAV9.hSYNI.hGBA prevented neuronal loss in treated *Gba* knock-out mice with neuron counts comparable to those of wild-type controls in key areas of the brain. This represents a significant improvement in what we have previously reported, where neonatal intravenous administration using a self-complementary vector with *GBA* gene under control of the ubiquitous beta-glucuronidase promoter (AAV9.GUSB.hGBA) did not provide complete neuroprotection. The AAV9.hSYNI.hGBA vector also carries the WPRE enhancing element downstream of the *hGBA* transgene, which is known to increase transgene expression of single-stranded AAV vectors when administered intravenously (22).

We previously demonstrated that intravenous administration of 4 × 10^11^ vg of AAV9.GUSB.hGBA to neonatal *Gba* knock-out mice resulted in significant increase in their lifespan but only partially ameliorated the neuropathology in P60 treated animals. In fact, despite the widespread overexpression of GCase throughout the brain, some areas were still affected by substantial neuroinflammation and neuropathology. With the aim of increasing neuronal transduction following systemic administration, we increased the dose of the new vector to 2.4 × 10^12^ vg (2.4 × 10^15^ vg/kg). Such high dose has not been used in the clinic yet, and might not be effectively translatable. However, the present work is a proof-of-concept study, aiming to demonstrate that a more efficient brain transduction is essential to increase the therapeutic outcome in the heavily affected brains of this acute mouse model.

Here we showed that the use of a neuron-specific vector completely normalized microglia activation in mice treated with AAV9.hSYNI.hGBA. This effect was especially evident in the S1BF cortical region, which was characterized by extensive microglia activation in mice injected with AAV9.GUSB.hGBA. The reduction of CD68-positive cells in AAV9.hSYNI.hGBA-treated mice was associated with normalization of neuron count in cortex, thalamus and cerebellum. These results supported the hypothesis according to which microglia activation temporally correlates with neuronal loss in defined brain regions (31). As a similar pathological pattern is observed in human patients, our findings might be an additional contribution to the development of a possible clinical intervention. The use of a neuron-targeted vector led to significant increase of GCase activity in the brain, although the enzyme levels were not completely normalized to wild-type levels. Nevertheless, even reduced levels of residual enzymatic activity can be sufficient to achieve significant therapeutic improvements. This hypothesis is sustained by the fact that heterozygote mice show lower GCase activity than wild-type in the brain and yet do not present neuropathology (25,20). Severe neuron loss and cerebral atrophy in the somatosensory region is characteristic of nGD. Reduction of cortical thickness was prevented in AAV9.hSYNI.hGBA-treated mice. In addition, cortical thinning is often accompanied by ventriculomegaly in different types of lysosomal storage disorders (32). No indication of pathological enlargement of the ventricles was present in treated mice. Together, our results demonstrated that preventing glucosylceramide accumulation in neurons rescued the acute and widespread neurodegeneration characteristic of neuronopathic Gaucher disease.

Administration of neuron-targeted gene therapy also led to normalization of astrogliosis in the cortex and thalamus, regions which contained considerable numbers of activated astrocytes in AAV9.GUSB.hGBA treated mice. Nevertheless, significant astrogliosis was detected in the hippocampal region CA1/CA2, cerebellum and the gigantocellular nuclei area of the brain stem of animals injected with AAV9.hSYNI.hGBA. Since we achieved complete neuroprotection in the areas examined and the microglial-mediated inflammatory response was prevented, it is possible that cross-correction to astrocytes is insufficient to prevent activation. However, this needs to be taken in the context that the role of astrocytes in nGD is not well understood and that the specific stimuli triggering neuroinflammation in lysosomal storage diseases have not been completely clarified.

Although human synapsin is a neuron-specific promoter, it has been shown that it drives expression in the liver following systemic administration in rodents (33). Evidence of low levels of vector-mediated gene expression was detected in the viscera of injected mice. Transduced liver cells might function as metabolic factory for the production of β-glucocerebrosidase. However, the effects of circulating enzyme were not sufficient to significantly ameliorate the pathology in all the visceral organs, particularly in the lung where tissue-resident macrophages (34) can still be impaired. Nevertheless, overall accumulation of Gaucher cells was partially reduced in 66-day-old treated mice compared to younger untreated knock-outs. Since visceral pathology is successfully improved by enzyme replacement therapy, the possible combination with neuron-targeted gene therapy might be considered as a beneficial treatment option for nGD patients.

Systemic delivery of AAV9 in two different nGD mouse models has been already attempted by Du and colleagues (35). Intravenous injection of an AAV9.CMV.Gba vector expressing the murine wild-type gene was administered 30 days before tamoxifen induction to Gba(flox/flox),UBC-CreERT2 mice, resulting in extension of the life span and increased GCase activity in the brain, liver and spleen of treated mice. An AAV9 vector with the synapsin promoter was also used to deliver the mouse *Gba* gene to P5 Gba(flox/flox), Nestin-Cre mice (25) via intraperitoneal injection. The treatment resulted in extended survival, amelioration of the neurodegeneration, but did not normalize enzymatic levels in the liver. Despite the overall positive results, the preventive or delayed AAV treatment might represent a possible therapeutic approach only for those type 3 patients displaying mild and late-onset neurological symptoms. However, we suggest that an early intervention is the only viable treatment option for type 2 patients, where the acute and lethal neurodegeneration is already present at birth (13).

Intravenous administration of AAV9 vectors is minimally invasive and results in broad transduction of the central nervous system. The recent clinical trial of gene therapy for spinal muscular atrophy further supports the efficacy and safety of this approach. However, we appreciate that the large dose of vector needed for a systemic intervention must be considered in terms of scalability and manufacturing costs. The possible immune response to high dose gene therapy must also be evaluated, although the SMA clinical trial showed that increased levels of transaminases in treated patients were successfully managed by prophylactic treatment with prednisolone.

The use of a single vector as unified treatment for the whole Gaucher patient population is an attractive therapeutic option. However, different vector configurations and novel engineered capsids can be considered in order to enhance transduction efficiency and specificity and possibly reduce the viral load. The AAV-PHP.B variants (36,37), for example, showed incredible CNS transduction efficiency when administered intravenously to rodents; however, as of today the superior characteristics of these new engineered capsids have not translated across different models and species (38). The use of strong ubiquitous promoters, enhanced synthetic promoters and optimized transgene sequences can improve gene delivery and expression in different organs, with greater therapeutic effects on both the neurological and visceral pathology.

In conclusion, our study validated neuron-targeted gene therapy as a potential option for the treatment of severe neurodegeneration in Gaucher disease, for which there is currently no cure. Nevertheless, we suggest that gene delivery limited to neurons only might not be sufficient in the long term.

## Materials

### Cloning

The *eGFP* gene and the human *hGBA* gene (NM_000157.4) were cloned into a single-stranded AAV vector under control of the human synapsin promoter hSynI. The enhancing WPRE sequence and the polyA sequence derived from the human growth hormone polyadenylation signal were cloned downstream. The expression cassette was flanked by AAV2 inverted terminal repeats.

### Transfections

HEK-293 T cells were transfected with 2 μg of DNA plasmid and 1 μL/μg DNA of polyethylenimine MAX (Polyscience, Nile IL, USA) in Opti-MEM (Gibco Thermo Fisher Scientific, Waltham MA, USA). At 48 h post-transfection, the cells were lysed with ice-cold RIPA Lysis buffer (Thermo Fisher Scientific, Waltham MA, USA) supplemented with 1X of protease inhibitor cocktail (Thermo Fisher Scientific). Cells were then harvested and centrifuged at 14 000 × g at 4°C for 15 min, and the supernatant was stored at—20°C for future use.

### Western blot analysis

Total protein concentration of samples was estimated using the Pierce BCA Protein Assay Kit Protein (Thermo Fisher Scientific) and normalized to 1, 0.5 and 0.1 μg/μL. 1X LDS Sample buffer (Life Technologies, Carlsbad CA, USA) and 1X Reducing agent (Life Technologies) were added to each sample. A total of 25, 12.5 and 2.5 μg of denatured protein sample and 10 μL of molecular weight marker (RPN800E, GE Healthcare, Chicago IL, USA) were separated via SDS-PAGE electrophoresis on NuPAGE Bis-Tris 4–12% polyacrylamide gel (Novex, Life Technologies). The samples were transferred to an equilibrated PDVF membrane (Millipore, Burlington MA, USA) at 400 mA for 1 h. The membrane was blocked in 5% BSA in TBS-Tween at 4°C for 1 h with agitation and consequently incubated with the primary antibody for GCase (1:1000, G4171, Sigma-Aldrich, St. Louis MI, USA) and actin (1:5000, AC-74, Sigma-Aldrich) diluted in TBS-Tween with 3% BSA at 4°C overnight with agitation. The membrane was then incubated with the secondary HRP-conjugated antibody goat anti-rabbit HRP (1:3000, ab6721, Abcam, Cambridge, UK) and goat anti-mouse HRP (1:2000, ab6789, Abcam) diluted in TBS-Tween with 3% BSA at room temperature for 1 h with agitation. The membrane was developed using SuperSignal West Pico kit (Thermo Fisher Thermo Fisher Scientific) accordingly to the manufacturer’s instructions and imaged using a InGenius imager (Syngene, Cambridge, UK).

### Virus production

HEK-293 T cells were transfected as described in (26) with the plasmid expressing the *hGBA* transgene, the packaging plasmid carrying the AAV9 *Cap* gene and the plasmid expressing the adenovirus helper genes in a 1:1:3 proportion. After 72 h, both the supernatant and cells were collected and processed. Purification of viral particles was performed via ultracentrifugation in increasing iodixanol (OptiPrep, Sigma-Aldrich) gradient for 3 h at 200 000 × g (ultracentrifuge tubes and SW40-Ti swinging-bucket rotor, Beckman Coulter, Brea CA, USA). The purified vector was extracted, resuspended in phosphate-buffered saline and filter sterilized (EMD Millipore Steritop, Thermo Fisher Scientific). The vector was concentrated (Vivaspin 20, 100 kDa cut off, Sartorius Stedim Biotech, Göttingen, Germany) and titered via alkaline gel electrophoresis and SDS-PAGE.

### *In vivo* animal studies

The K14-Cre colony was maintained as heterozygous. Pups were genotyped at birth through GCase enzymatic assay on dried blood spot samples. Blood samples were taken from the temporal vein and blotted on filter paper (Whatman 903 CDC 5-spot card 100/pk, GE Healthcare). A 6 mm disc was punched from the centre of the blood spot and incubated in a 0.2 M citrate buffer with 10% Triton X-100 (Sigma-Aldrich) and sodium taurodeoxycholate (Sigma-Alrich) for 1 h at 4°C in constant agitation. The samples were then incubated with the substrate 4-methylumbelliferyl-β-D-glucopyranoside (Sigma-Alrich) at 37°C overnight. The reaction was stopped by adding 0.5 M EDTA to the samples the following day. Serial dilutions of 4-methylumbelliferone (Sigma-Aldrich) were used as standards. Fluorescence was read using FluoStar Optima Plate Reader (BMG Labtech, Aylesbury, UK).

Wild-type CD1 strain mice were used in the eGFP reporter study and in the AAV9.hSYNI.hGBA neurotoxicity study.

At post-natal day 1, pups were anaesthetized on ice for 30 s and injected via the superficial temporal vein with 2 × 10^11^ viral vector genomes of AAV9.hSYNI.eGFP or 2.4 × 10^12^ viral vector genomes of AAV9.hSYNI.hGBA. Fully recovered pups were returned to the dam cage.

Mice were euthanized by transcardial perfusion using phosphate-buffered saline while under terminal isoflurane anaesthesia and organs were harvested.

All listed procedures were conducted following the Animal Research Reporting of *In Vivo* Experiments and were approved by the UK Home Office for the conduct of regulated procedures under license (Animal Scientific Procedure Act, 1986) and by the ethical review committees of University College London. The Animal Research Reporting of *In Vivo* Experiments (ARRIVE) guidelines from the National Centre for the Replacement Refinement and Reduction of Animals in Research were followed.

### Animal behavioural analysis

#### Rotarod test

Mice were trained for 3 days before performing the tests. The rotarod (Panlab LE8200, Cornella, Spain) was set with a start speed of 4 rpm and 20 rpm/min acceleration. When a mouse fell off the time and speed were noted, up to three times per experiment. If the mouse fell within the first 5 s due to poor placing, the experiments would not be recorded. 

#### Open field test

The mouse was placed in the centre of a square transparent Plexiglas chamber measuring 27 cm × 27 cm and allowed to freely explore the chamber. Animals were filmed from the top of the chamber. The duration of each session was 4 min. The analysis of the tests was carried out using ANY-maze Behaviour Tracking Software v. 4.99 (Stoelting, Dublin, Ireland), assessing distance, average speed, mobility and immobility time of each animal.

#### Hind limb clasping test

Hind limb clasping phenotype was evaluated following the protocol described in (39). The mouse was suspended by the end of its tail approximately 20 cm from the bench, ensuring that the animal cannot grasp its tail. The test was recorded for 1 min with a camera facing towards the abdomen of the mouse. The clasping phenotype was scored as following: all limbs extended out from the body all the time = 0; hind limbs extended out most of the time; one hind limb retracted in a clasping position less than 50% of the time = 1; both hind limbs partially retracted for more than 50% of the time = 2; hind limbs exhibit a clear clasping phenotype touching the abdomen for more than 50% of the time = 3.

#### Righting reflex test

The mouse was placed in a supine position on a flat surface and rapidly released. The ability of the mouse to right itself (four paws on the ground) was assessed. Fast latency to return to prone position (<1 s) was scored 1, while animals with impairments in motor coordination functions and slow latency to right their selves (>10 s) were scored 0.

#### Foot fault test

The mouse was placed on a metal mesh surface (mesh gap size 1.3 cm) elevated 20 cm from the bench. The animal was allowed to cross the device from a ‘start cage’ to the ‘home cage’. The run was recorded over 1 min. The number of total steps for fore limbs and hind limbs was manually counted. The number of foot faults, defined as misplacement of a paw slipping through the grid, was also manually counted.

#### Footprint pattern test

The paws of the mouse were painted with different coloured non-toxic dyes (fore paws: blue; hind paws: red). The animal was then allowed to walk along a corridor 10 cm wide, leaving a trail of footprints on a paper sheet 60 cm long. The mouse was trained to spontaneously walk through the corridor for three times before recording the experiment. The stride length and base width were manually measured.

## Histological Analysis

Fixed organs were frozen and cut at 40 μm in thickness at a constant temperature of −20°C with a Cryostat Leica CM3050 (Leica Biosystems, Milton Keynes, UK).

A series of representative sections were collected and washed with 1X tris-buffered saline (TBS). Endogenous peroxidase activity was blocked with 1% H_2_O_2_ in 1X TBS for 30–60 min under constant gentle agitation. Slices were rinsed in TBS and the non-specific binding was blocked in 15% normal serum (Sigma-Aldrich) in TBS-T (0.3% Triton X-100 in 1X TBS) for 30 min in agitation. Sections were incubated at 4°C overnight with the primary antibody for eGFP (1:1000, ab290, Abcam), GCase (1:1000, G4171, Sigma-Aldrich), CD68 (1:2000, MCA1957, AbD Serotech, Kidlington, UK), GFAP (1:1000, MAB3402, Millipore), LAMP1 (1:2000, ab24170, Abcam), calbindin (1:10 000, CB38, Swat, Marly, Switzerland) diluted with 10% normal serum in TBS-T. The following day, slices were washed with 1X TBS and incubated for 2 h at room temperature with the secondary biotinilated antibody anti-mouse, anti-rabbit or anti-rat IgG (1:1000, Vector Laboratories Inc., Burlingame CA, USA) diluted with 10% normal serum in TBS-T. Afterwards, sections were incubated for 2 h with 1:1000 avidine-biotin reagent (Vectastain Elite ABC kit, Vector Laboratories) and immunoreaction was detected by adding 0.05% 3,3′-diaminobenzidine tetrahydrochloride (Sigma-Aldrich). Sections were mounted on chrome-gelatine coated slides, dehydrated in 100% ethanol, cleared in Histo-clear (National Diagnostic, Atlanta GA, USA) and cover slipped with DPX mountant (Thermo Fisher Scientific).

Immunofluorescent staining of tissue sections was performed by incubating the sections with primary antibodies for CD68 (1:100, MCA1957, AbD Serotech), GFAP (1:200, MAB3402, Millipore) and NeuN (1:500, MAB377, Millipore); secondary antibodies Alexa488 (A11008, Life Technology) and Alexa568 (A11034, Life Technology) and counterstaining with DAPI. Sections were mounted on chrome-gelatine coated slides and coverslipped with Fluoromount G (SouthernBiotech, Birmingham AL, USA).

For the haematoxylin and eosin staining, tissue sections were mounted on chrome-gelatine coated slides and air-dried overnight. Sections were then stained protected from light with filtered 0.1% Mayer Haematoxylin (Sigma-Aldrich) for 10 min. The slides were rinsed in distilled water and consequently incubated in 0.5% eosin solution (Sigma-Aldrich). The sections were quickly washed and subsequently dehydrated for 30 s in rising concentrations of ethanol (50, 70, 95, 100%). The slides were finally incubated in Histo-clear for 30 min and coverslipped with DPX mountant medium.

Light bright-field images were taken with a Nikon DS-Fi1 camera (Nikon, Tokyo, Japan) attached to a Nikon Eclipse E600 microscope. Representative images of discrete areas of the sections were taken with 10X/0.25 and 40X/0.65 objectives (CFI Achromat, Nikon).

Immunofluorescent stained sections were analysed with Zeiss LSM 710 laser scanning confocal microscope (Carl Zeiss AG, Oberkochen, Germany).

## Staining Quantification

The quantification of immunohistochemical staining was conducted as described in (20). Briefly, 10 consecutive non-overlapped images per distinct area were taken with constant light intensity. The quantification of the immunoreactivity of stained sections was performed using the Image-Pro Premier analysis system (Media Cybernetics, Rockville MD, USA). The minimum threshold value of stained pixel intensity was assigned for all the images of sections stained with the same antibody. The extent of staining, defined as level of intensity above the assigned threshold for each stained pixel and expressed as percentage of the total area of the image, was measured. Results were presented as the percentage of average value of immunoreactivity of 10 consecutive images for each distinct region.

## Stereology

Brain sections were mounted on chrome-gelatine coated slides and air-dried overnight. Slides were stained in 0.05% Cresyl Violet solution (VWR, Radnor PA, USA) at 60°C for 30 min. Slides were then rinsed twice in fresh distilled water and dehydrated in increasing concentration of IMS. Sections were then incubated in fresh Histo-clear for 30 min and consequently coverslipped with DPX mountant medium.

Neuron counting and cortical thickness measurements were estimated with Stereo Investigator software (MBF Bioscience, Williston VE, USA) on Nissl stained sections with a Nikon Optihot light microscope (Nikon) attached to a Q-Imagin camera (2000R-CLR-12, MBF Bioscience).

Neurons were counted with the optical fractionator probe using the 40X objective. The grid size and the counting frame used for analysing different brain regions were the following: S1BF 150 × 150 μm, 50 × 50 μm; VPM/VPL 175 × 175 μm, 50 × 50 μm; Gi 100 × 100 μm, 50 × 50 μm. Cells were counted using a 100X objective. Efficient sampling was estimated by a coefficient of error between 0.05 and 0.1 (40).

The mean thickness of the S1BF cortical region was estimated by using the Cavalieri vertical sections principle (41). The length of 10 parallel consecutive lines intersecting perpendicularly the cerebral cortex, traced from the somatosensory barrel cortical layer 1 to the corpus callosum, was measured.

### Enzymatic assay

Frozen samples were first homogenized in distilled water and consequentially incubated with 4.8 mM 4-methylumbelliferyl-β-D-glucopyranoside (Sigma-Alrich) in 0.15 M citrate/phosphate buffer pH 5.1 containing 1.5% sodium taurodeoxycholate (Sigma-Aldrich) at 37°C for 2 h. The reaction was stopped with 1 M glycine buffer pH 10.4. Fluorescence of the samples and 4-methylumbelliferone standard was read with FluoStar Optima Plate Reader (BMG Labtech, Aylesbury, UK). The enzymatic activity was calculated and expressed in nmol/hr/μg.

### Glycosphingolipid profile analysis

Glycosphingolipids (GSLs) from brain homogenates were analysed as described in (20). Briefly, GSLs were extracted in chloroform:methanol (1:2 v/v) overnight at 4°C and then purified using solid phase hydrophobic C18 chromatography columns (Kinesis, Neots, UK). The GSLs were then digested overnight to release glycan headgroups with recombinant Endoglycoceramidase I (gifted from Orphazyme), or with Cerezyme (Sanofi Genzyme) to release glucose from glucosyl-ceramide. Liberated glycans and glucose were then labelled with anthranilic acid (2-AA). The resulting 2AA-labelled glycans were purified and separated from the fluorescent label using Supelco DPA-6S solid phase extraction columns (Sigma-Aldrich) and then applied to normal-phase high-performance liquid chromatography (NP-HPLC). Integrated peaks from the chromatogram were quantified using a 2AA-labelled chitotriose standard and normalized to total protein content.

### Statistics

The statistical analysis was performed with GraphPad Prism Software (v. 6.0e). Data are presented with indication of average values, and standard deviation (SD) was used as variation value. t-test, one-way ANOVA and two-way ANOVA tests were performed where appropriate. The survival data were analysed with Kaplan–Meier estimate. Tukey’s and Mantel-Cox’s honest significance *ad hoc* tests were used for multiple comparison analysis. Non-statistical significance (ns) was noted for *P* > 0.05.

## Author Contributions

G.M. contributed to scientific investigation, analysis of the data and manuscript drafting. M.P.H. and S.M.W. contributed to the acquisition of the *in vivo* data and manuscript revision. KL.W., D.A.P. and F.M.P. contributed to lipid analysis and manuscript revision. S.N.W. and A.A.R. contributed to experimental design and manuscript revision.

*Conflict of Interest statement.* None declared.

## Funding

This work was funded by the UK Gauchers Association and UCL IMPACT Studentship. KL.W. received funding from UK Medical Research Council (Gaucherite grant reference: MR/K015338/1). F.M.P. is a Royal Society Wolfson Research Merit Award and a Wellcome Trust Investigator in Science. S.N.W. received funding from UK Medical Research Council grants; G1000709 and MR/N026101/1, MR/R015325/1, MR/P026494/1 and MR/N019075/1, and from SPARKS 17UCL01. A.A.R. receives funding from the UK Medical Research Council (MR/R025134/1, MR/R015325/1, MR/N026101/1, MR/S009434/1), Action Medical Research (GN2485) and Asociación Niemann Pick de Fuenlabrada.
